# Two‐day low‐dose dexamethasone suppression test more accurate than overnight 1‐mg in women taking oral contraceptives

**DOI:** 10.1002/edm2.255

**Published:** 2021-05-26

**Authors:** Tiphaine Carton, Elise Mathieu, Fleur Wolff, Jason Bouziotis, Bernard Corvilain, Natacha Driessens

**Affiliations:** ^1^ Department of Endocrinology Cliniques Universitaires de Bruxelles Hôpital Erasme Université Libre de Bruxelles (ULB) Brussels Belgium; ^2^ Department of Clinical Chemistry Laboratoire Hospitalier Universitaire de Bruxelles (LHUB‐ULB) Université Libre de Bruxelles (ULB) Brussels Belgium; ^3^ Service de la Recherche Biomédicale Cliniques Universitaires de Bruxelles Hôpital Erasme Université Libre de Bruxelles (ULB) Brussels Belgium

**Keywords:** Cushing's syndrome, dexamethasone suppression test, false positive, oral contraception

## Abstract

**Introduction:**

Late‐night salivary cortisol (LSaC) and 24‐h urinary free cortisol measurement, and overnight 1‐mg dexamethasone suppression test (1 mg‐DST) are the first‐line screening tests recommended for Cushing's syndrome. Through elevations in the level of cortisol‐binding globulin, oral contraceptive agents lead to increases in the total plasma cortisol concentration, yielding false‐positive 1 mg‐DST results.

**Objective:**

To compare the accuracy of the overnight 1‐mg DST and two‐day low‐dose DST (2d‐DST) in female volunteers taking combined oestrogen‐progestin oral contraceptives (COCs).

**Methods:**

This prospective study enrolled 30 healthy participants. Their plasma cortisol response levels were compared after the 1‐mg DST and 2d‐DST and classified into three categories: normal (≤50 nmol/L), doubtful (51–138 nmol/L) and abnormal (>138 nmol/L). Salivary cortisol was also measured at late night and after the DSTs.

**Results:**

Following the 1‐mg DST and 2d‐DST, the plasma cortisol concentrations decreased to a median of 69 nmol/L and 37 nmol/L, respectively (*p* < 0.001). A statistically significant higher proportion of unclear or abnormal results were observed after the 1‐mg DST (63%) than after the 2d‐DST (27%) (*p* = 0.004). None of the values were >138 nmol/L after the 2d‐DST, while 11% of them were abnormal after the 1‐mg DST (*p* = 0.25). No LSaC value was abnormal.

**Conclusion:**

Our results suggest that, when late‐night salivary cortisol is not available, the 2d‐DST could be a better screening option than the 1‐mg DST for women taking oral contraceptive agents who are reluctant to stop them. This finding requires confirmation in those with a suspicion of hypercortisolism.

## Graphical abstract

This study compared the accuracy of the overnight 1‐mg dexamethasone suppression test (1 mg‐DST) and two‐day low‐dose DST (2d‐DST) in female volunteers taking combined oestrogen‐progestin oral contraceptives (COCs). Our results confirm that in women taking oral contraceptives, 1‐mg DST is not accurate as first‐line screening test. Our results suggest that, when late‐night salivary cortisol is not available, the 2d‐DST could be a better screening option than the 1 mg‐DST for women using oral contraceptive agents who are reluctant to stop them. The cessation of oral contraception may not always be mandatory for the performance of the 2d‐DST, particularly if the initial cortisol concentration is lower than 900 nmol/L.

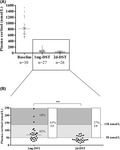

## 1 INTRODUCTION

The global increase in the prevalence of adrenal incidentaloma, obesity and diabetes mellitus has highlighted the requirement for techniques to rule out hypercortisolism.^1^, ^2^, ^3^, ^4^ The diagnosis of this condition, also known as Cushing's syndrome, remains challenging, as many of its associated symptoms and signs are nonspecific, particularly in its subclinical form.^5^ Nevertheless, even subclinical hypercortisolism is related to excess cardiovascular mortality, underlying the importance of its recognition and management.^6^, ^7^

Urine free cortisol (UFC) and late‐night salivary cortisol (LSaC) measurement, the overnight 1‐mg dexamethasone suppression test (1 mg‐DST), and the 2‐mg 48‐h DST (2d‐DST) are the classical first‐line screening tests in such settings^8^, ^9^; however, these techniques have both advantages and disadvantages. The choice of testing method employed depends on the accuracy of laboratory assays, which may depend on pathological aetiology and population type, as well as its availability. Of the 1‐mg DST, the 2d‐DST, UFC, LSaC and midnight serum cortisol measurement, and the dexamethasone‐suppressed corticotropin‐releasing hormone and desmopressin tests, 1 mg‐DST shows the strongest sensitivity and UFC measurement is less sensitive in Cushing's syndrome diagnosis.^10^

Women taking oral contraceptive agents have higher total plasma cortisol concentrations owing to elevations in the levels of cortisol‐binding globulin (CBG) and the stronger affinity to cortisol.^8^, ^11^, ^12^, ^13^, ^14^ Therefore, in patients with an elevated CBG level, the measurement of the total plasma cortisol level may yield imprecise findings in the assessment of plasma free cortisol, resulting in a high rate of false positive (FP) results in the 1 mg‐DST.^15^, ^16^, ^17^ Indeed, among women using oral contraceptives, the 1 mg‐DST FP rate is evaluated to be 50%.^8^, ^18^ Increases in the rate of CBG synthesis are associated with both the dose and duration of contraceptive use.

In 2015, according to the United Nations, 64% of married or in‐union women of reproductive age worldwide were using some form of contraception. Of the modern contraceptive methods, the usage rate of combined oestrogen‐progestin oral contraceptives (COCs) is 15% in developed countries. More than 100 million women, globally, use COCs.^19^

In clinical practice, the exclusion of Cushing's syndrome could also be a requirement among women using oral contraceptives. In such patients, clinicians mandate the cessation of contraceptive use for 6 weeks before 1 mg‐DST performance.^8^

The sensitivity and specificity of the 1 mg‐DST (overnight 1 mg dexamethasone) and 2d‐DST (0.5 mg dexamethasone every 6 h for 48 h) are considered equal; however, the 2d‐DST is less frequently used in ruling out Cushing's syndrome.^20^ To the best of our knowledge, no study till date has compared the sensitivity and specificity of these two tests among women using oral contraceptives.

Therefore, this study aimed to compare the efficacy of the overnight 1‐mg DST and two‐day low‐dose DST in healthy volunteers using oral contraceptives.

## 2 MATERIALS AND METHODS

This prospective clinical study was approved on January 16, 2018 by the Ethics Committee on Research Involving Human Subjects of the CUB Hôpital Erasme‐ULB (Erasmus P2017/405 and CWB B406201733024) and was conducted from January 23, 2018 to April 3, 2018. Written consent has been obtained from each subject after full explanation of the purpose and nature of all procedures used.

We enrolled 30 healthy female volunteers, aged between 18 and 45 years, who were taking COCs for at least 6 months. The exclusion criteria were the presence of signs or symptoms of hypercortisolism, a body mass index (BMI) higher than 30 kg/m^2^, and the use of medications that potentially interfere with cortisol metabolism. Subclinical hypercortisolism was excluded using LSaC measurements that were performed among all the volunteers. Four participants opted out of the study for personal reasons.

Each participant underwent an overnight 1‐mg DST and a 2d low‐dose DST (intake of 0.5 mg dexamethasone every 6 h for 48 h) with a 1‐week break, at minimum, between the two tests. The study was designed such that the participants visited the clinic on three mornings: the first visit corresponded to the measurement of the basal cortisol value, the second to the evaluation of 1 mg‐DST cortisol response, and the third to the performance of the 2d‐DST. The levels of morning adrenocorticotropic hormone (ACTH) and salivary cortisol were determined before and after the DSTs and those of CBG before the DSTs.

The level of serum cortisol was measured using competitive electrochemiluminescent tests on Cobas E (kit cortisol II for serum cortisol and cortisol kit for urinary cortisol after liquid‐liquid extraction, Roche diagnostics, Vilvoorde, Belgium). The level of ACTH was quantified using an electrochemiluminescent sandwich test on Cobas E (Kit ACTH, Roche Diagnostics, Vilvoorde, Belgium), while that of serum CBG was quantified using a competitive radio‐immunological test (Kit CBG, ZenTech, Liège, Belgium). Saliva sampling was performed using Salivettes® with a synthetic Sarstedt‐branded swab. The level of salivary cortisol was measured after separation by high‐performance liquid chromatography (HPLC 1260 Infinity, Agilent Technologies, Diegem, Belgium) using tandem mass spectrometry (6490 triple Quadrupole, Agilent Technologies, Diegem, Belgium).

All statistical analyses were conducted using the Stata/IC 15.1 software. The normality of the distributions of the quantitative data was evaluated with graphical representations (histogram, box plot, q‐q plot). The mean and standard deviation (SD) were used to describe symmetrical distributions, while the median and interquartile range (IQR) were used for asymmetrical distributions. As the differences between the cortisol values were not normally distributed, a Wilcoxon signed‐rank test was used to compare the values between the two tests. The Friedman test was also used for the analysis of repeated measures. McNemar's exact test was used to compare the proportions of the categorical outcomes between the 1 mg‐DST and 2d‐DST. The Mann‐Whitney‐Wilcoxon test was conducted for the comparison of data between the independent subgroups. Spearman's correlation coefficient (*r*_s_) was used for the measurement of the correlation between the parameters. Logistic regression and repeated measures logistic regression were performed to determine the factors that were associated with the 1 mg‐DST and 2d‐DST outcomes.

## 3 RESULTS

**FIGURE 1:**
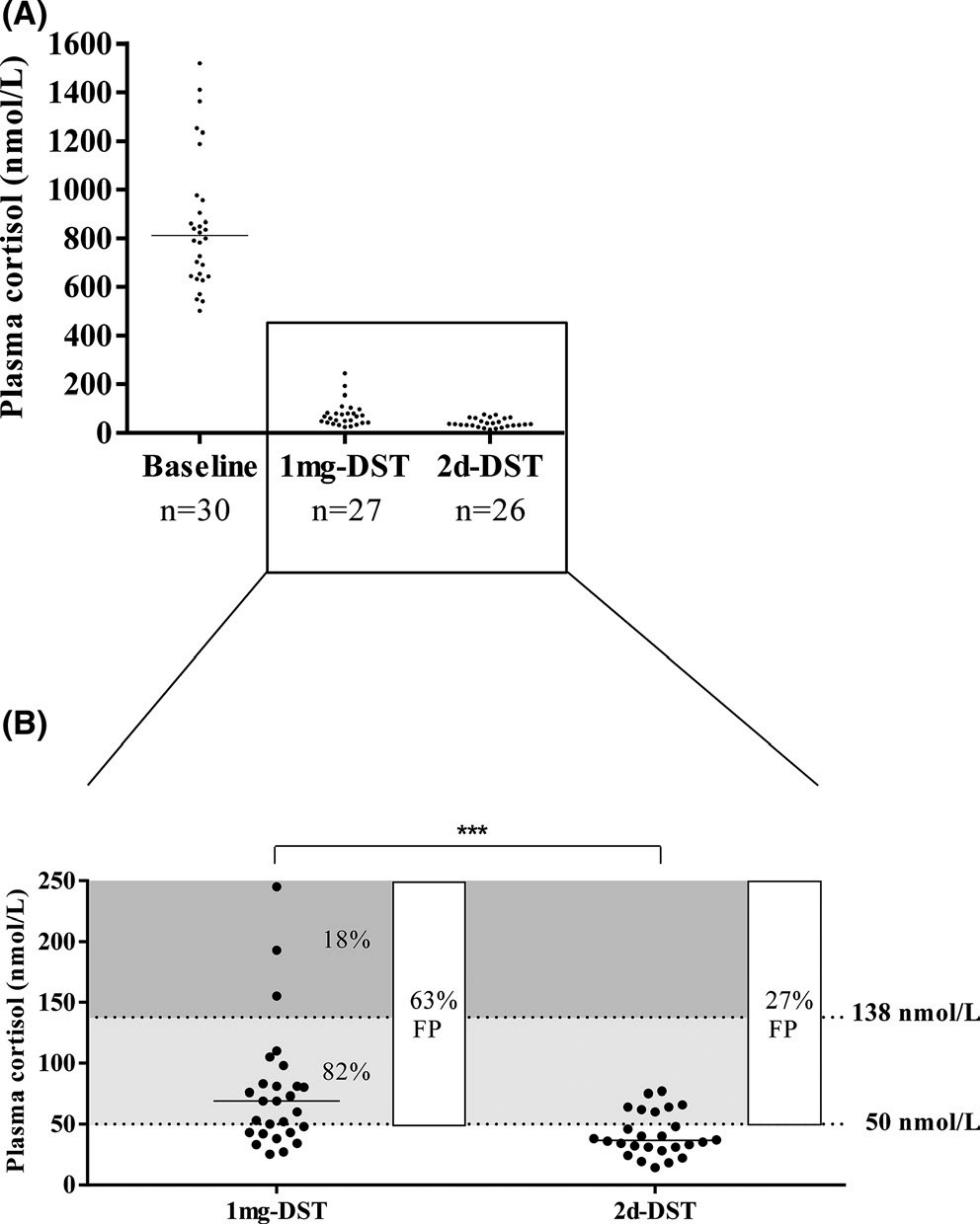
A, Morning plasma cortisol level at the baseline and after the 1 mg‐DST and 2d‐DST. Each point represents the morning plasma cortisol level at the baseline and after the 1 mg‐DST and 2d‐DST of one healthy volunteer. The baseline median value is represented by the horizontal bar on the box plot. The difference between the baseline values and those following the test between the 1 mg‐DST (*p* < 0.001) and 2d‐DST (*p* < 0.001) were statistically significant; the overall difference in the repeated measures analysis was also significant (*p* < 0.001). DST, dexamethasone suppression test. B, Cortisol response after the 1 mg‐DST and 2d‐DST. Each point represents the plasma cortisol value after the 1 mg‐DST and 2d‐DST of one healthy participant. The median values are represented by the horizontal bars on the scatter dot plot. The shaded square represents the rate of FPs in the DSTs: doubtful: between 51 and 138 nmol/L and abnormal >138 nmol/L. Totally, an FP rate of 63.0% (17/27) is shown to be distributed in the two zones (82.3% in the doubtful and 17.7% in the abnormal zones) after the 1 mg‐DST and a corresponding rate of 26.9% (7/26) is shown exclusively in the doubtful zone after the 2d‐DST. This difference in the FP rate is statistically significant, at *p* = 0.004 (***). DST, dexamethasone suppression test; FP, false positive

A total of 26 volunteers completed the study, following attendance at the final visit. In our sample, the median (IQR) age was 25 (24–27) years, median (IQR) BMI was 21.1 (19.6–22) kg/m^2^, median (IQR) ethinyl estradiol dosage was 20 (20–30) µg, and COC intake duration was 8.0 ± 2.8 years (mean ± SD). At the baseline, the median (IQR) morning plasma cortisol level was 812.5 (644–957) nmol/L (min‐max values: 503–1521; *n* = 30); this decreased to median (IQR; min‐max value) levels of 69 (43–83; 25–245) nmol/L and 36.5 (31–60; 14–77) nmol/L following the 1 mg‐DST (*n* = 27) and 2d‐DST (*n* = 26), respectively (Friedman's test *p* < 0.001, Wilcoxon signed‐rank test for the comparison of the measures between the 1 mg‐DST and 2d‐DST, *p* < 0.001) (Figure 1A). At the baseline, median (IQR) ACTH concentration was 21.7 ng/L (15.4–33.2; *n* = 30) and decreased after both tests below the lower limit detection of our assay.

The responses to the plasma cortisol suppression tests were classified into normal (≤50 nmol/L), doubtful (51–138 nmol/L) and abnormal (>138 nmol/L), with the two last corresponding to FP results. The FP rates were 63% and 27% after the 1 mg‐DST and 2d‐DST (*p* = 0.004), respectively (Figure 1B). However, none of the values was higher than 138 nmol/L after the 2d‐DST; 11% of the values were found to be abnormal after the 1 mg‐DST. Indeed, all the FPs observed after the 2d‐DST were in the doubtful zone and the maximum plasma cortisol level was 77 nmol/L.

**FIGURE 2:**
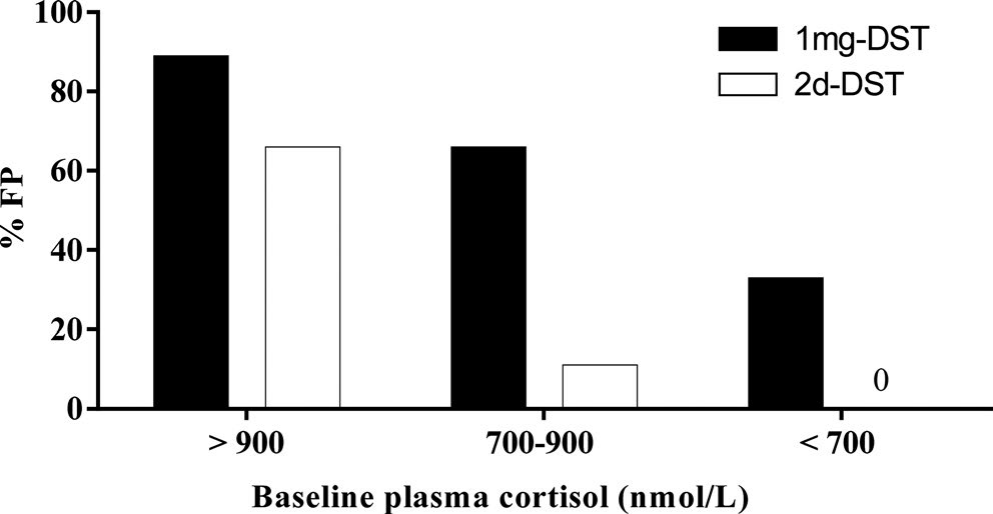
Rate of false positive results in the 1 mg‐DST and 2d‐DST in association with the baseline plasma cortisol value. The false positive percentage in association with each DST (black bar for the 1 mg‐DST and white bar for the 2d‐DST) is expressed in relation to the plasma cortisol level at the baseline in the studied population, which was divided into tertiles: one‐third of them had an initial plasma cortisol level <700 nmol/L, approximately another third (36.7%) had a level between 700 and 900 nmol/L, and one‐third (30.0%) had a level >900 nmol/L. DST, dexamethasone suppression test; FP, false positive

We analysed the FP rate in relation to the initial cortisol concentration through the division of the population into tertiles based on the baseline cortisol concentration: one‐third of the population had a basal plasma cortisol level <700 nmol/L, approximately one‐third (36.7%) had a level between 700 and 900 nmol/L, and the final third (30%) had levels >900 nmol/L. In participants with a baseline plasma cortisol level ≤900 nmol/L (first and second tertiles), the FP rate was significantly lower after the 2d‐DST than after the 1 mg‐DST (6% vs. 50%, *p* = 0.02). The FP rate difference was greater in the two tertiles with the lowest initial cortisol value (Figure 2). The degree of reduction in the level of cortisol (expressed as the percentage decrease in the level from that at the baseline) was statistically higher (*p* < 0.001) after the 2d‐DST (median [IQR] decrease: 96 [92–98] %) than after the 1 mg‐DST (median [IQR] decrease: 92 [84–96] %). The degree of reduction in the cortisol level from that at the baseline was also statistically higher among those with a normal response than in the women with a doubtful or an abnormal response (FP) both after the 1 mg‐DST (*p* < 0.001) and 2d‐DST (*p* = 0.03).

**FIGURE 3:**
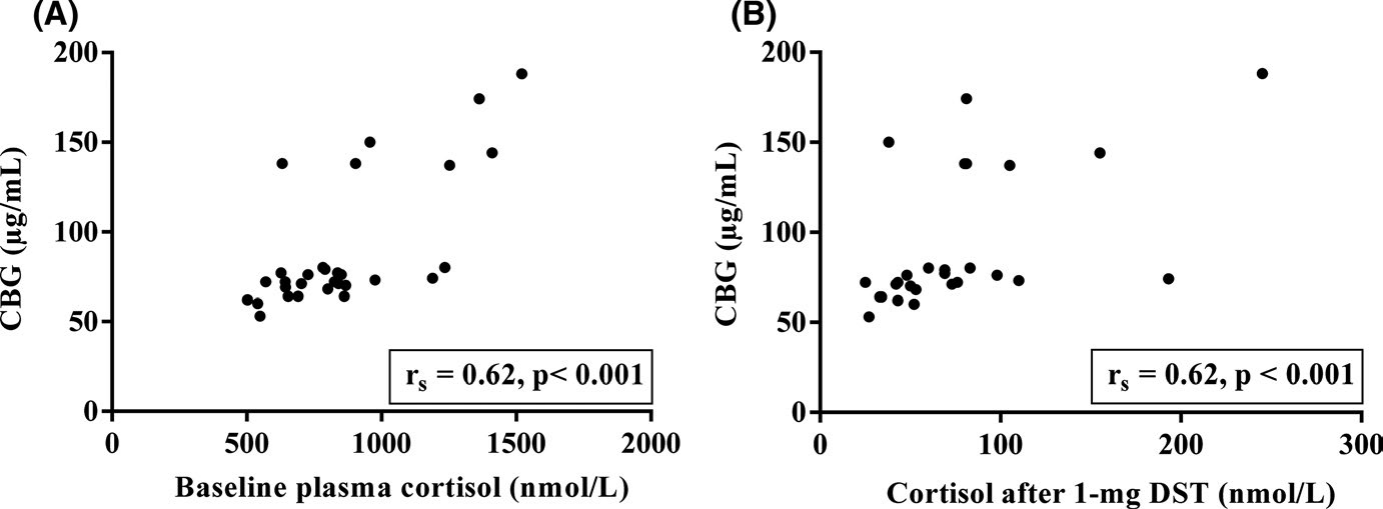
Correlation between CBG and plasma morning cortisol at baseline (A) and after 1 mg‐DST (B). The figure represents the significant positive correlation between the level of CBG and plasma morning cortisol at baseline (A) and after 1 mg‐DST (B). CBG, cortisol binding globulin; DST, dexamethasone suppression test

As expected, there existed a significantly positive correlation between the levels of CBG and morning plasma cortisol at baseline (*r*_s_ = 0.62, *p* < 0.001) as well as morning plasma cortisol after 1 mg‐DST (*r*_s_ = 0.62, *p* < 0.001) (Figure 3). The median (IQR) CBG concentration was 73.5 (69–80) µg/ml at the baseline (*n* = 30). All volunteers had LSaC levels in the normal range (lower than 2.8 nmol/L) and morning saliva cortisol levels lower than 1.9 nmol/L after both the 1 mg‐DST (*n* = 27) and 2d‐DST (*n* = 26) (Table 1). We did not observe any correlation (*r*_s_ = −0.03, *p* = 0.88) between CBG and basal morning salivary cortisol (median [IQR] 3.40 [6.41–15]; *n* = 30).

**TABLE 1:** Individual response after DSTs

# participant	Baseline plasma cortisol (nmol/L)	Plasma cortisol after 1 mg‐DST (nmol/L)	Plasma cortisol after 2d‐DST (nmol/L)	Baseline morning salivary cortisol (nmol/L)	Late‐night salivary cortisol (nmol/L)	Morning salivary cortisol after DSTs (nmol/L)
16	503	43	31	6.5	<1.9	<1.9
4	540	52	40	11.0	<1.9	<1.9
3	549	27	18	15.2	<1.9	<1.9
9	570	25	19	7.0	<1.9	<1.9
20	627	69	**–**	13.4	<1.9	<1.9
26	632	81	40	15.0	<1.9	<1.9
2	643	43	22	6.4	<1.9	<1.9
10	644	**–**	**–**	4.9	**–**	**–**
15	654	33	33	15.3	<1.9	<1.9
11	691	34	24	16.4	<1.9	<1.9
21	703	73	32	5.2	2.1	<1.9
7	727	48	34	7.1	<1.9	<1.9
13	783	60	14	6.4	<1.9	<1.9
1	791	69	46	7.8	<1.9	<1.9
28	800	53	28	9.9	<1.9	<1.9
30	825	76	35	9.3	<1.9	<1.9
24	837	**–**	**–**	6.2	**–**	**–**
17	840	42	38	7.6	<1.9	<1.9
18	850	98	75	5.2	<1.9	<1.9
8	862	**–**	**–**	25.0	**–**	**–**
25	867	50	37	**–**	<1.9	<1.9
23	904	80	62	9.5	<1.9	<1.9
19	957	38	36	5.5	<1.9	<1.9
12	976	110	64	15.4	<1.9	<1.9
27	1189	193	31	14.4	<1.9	<1.9
22	1235	83	48	8.3	<1.9	<1.9
5	1253	105	77	18.1	<1.9	<1.9
29	1363	81	66	9.5	<1.9	<1.9
6	1411	155	60	19.4	2.8	<1.9
14	1521	245	64	10.0	<1.9	<1.9

Note

Table represents the morning plasma and salivary cortisol value at the baseline and after DSTs and the late‐night salivary cortisol before DST of each healthy participant. Doubtful values are represented in light grey (cortisol between 51 and 138 nmol/L after DSTs) and abnormal response in dark grey (cortisol > 138 nmol/L after DSTs). In terms of the rate of FPs after the 1 mg‐DST, we observed recovery to normal response in more than half (56.3%) of the participants after the 2d‐DST. Salivary cortisol was in normal range below 18.4 nmol/L in morning time and below 2.8 nmol/L at late night.

Abbreviations: DST, dexamethasone suppression test; FP, false positive.

Since we do not have a group suspected of hypercortisolism in our study, we can only calculate the specificity of the DSTs. The specificity of the 1‐mg DST was 10/27 = 37.0 [19.4–57.6] % and the specificity of the 2d‐DST was 19/29 = 73.1 [52.2–88.4] %.

Median (IQR) time interval between the two tests was 15 days (12–23; *n* = 26) with no significant differences according to the observed type of response [16 days (12–21) in case of normal response after both 1 mg‐DST and 2d‐DST, 15 days (10–22) in case of FP after 1 mg‐DST and normal after 2d‐DST and 15 days (13–33) in case of FP after either 1 mg‐DST and 2d‐DST] (Table 1).

Logistic regression was performed to determine whether age, BMI, COC type, estradiol dosage, OPC use duration, initial CBG concentration and initial plasma cortisol concentration had the potential to predict the DST response type (normal or FP). The COC types (depending on the progestogen type) were second‐generation COC with levonorgestrel (*n* = 5), third‐generation COC with desogestrel or gestoden (*n* = 13), and other COCs with dienogest, drospirenone, chlormadinone acetate or cyproterone acetate (defined as the anti‐androgenic COC group; *n* = 12). We separately investigated the associations between the aforementioned parameters and the outcomes following both tests. We then constructed a multivariable model accounting for repeated measures, from which adjusted odd ratios were derived. Greater initial CBG concentrations were associated with FP outcomes after the 2d‐DST test (odds ratio [OR] 1.04, 95% confidence interval [CI] 1.01–1.07) but were not included in the final model. The final model included only the initial plasma cortisol concentration and COC type. A baseline cortisol concentration >900 nmol/L was associated with FP outcomes both after the 1 mg‐DST and 2d‐DST (OR 5.21, 95% CI 1.27–21.37). The odds of having an FP result was approximately four times lower when third‐generation COCs were used compared to that associated with anti‐androgenic COC use (OR 0.24, 95% CI 0.06–0.97). In terms of the other parameters, no statistically significant association was observed.

## 4 DISCUSSION

In this study, we compared the accuracy of the overnight 1‐mg and 2d low‐dose DSTs in volunteers taking oral contraceptive agents. This study was justified by the fact that, in women using oral contraception, the 1 mg‐DST FP rate is not lower than 50%.^8^

In our study population, an FP rate of 63% was observed after the 1 mg‐DST; this value is comparable to that noted in other studies. Vastbinder et al. reported an FP rate of 62% in their study, which prospectively investigated the efficacy of the 1 mg‐DST in a healthy volunteer population using oral contraceptives (18). In this study, the FP rates were compared after the discontinuation of oral contraceptive use either for a short duration (1 week) or longer durations (6 weeks). A significant decrease was observed in the degree of cortisol suppression following the 1 mg‐DST (62% to 8%; *p* < 0.02) after a short 1‐week interruption of oestrogen‐progestin use; no FPs were observed after 6 weeks of COC discontinuation.^18^

Even if the simplest way to avoid a false positive test is to stop the COC before a DST, some women prefer to avoid an interruption of their COC for personal reasons. We evaluated in volunteers whether the 2d‐DST is more accurate than the 1 mg‐DST to exclude hypercortisolism in women taking COC and therefore to avoid having to interrupt COC in all women. As suggested by the most recently published guidelines on the management of adrenal incidentaloma, which recommend 1 mg‐DST performance, we categorized the DST responses into three subgroups: normal, intermediate and frankly altered cortisol suppression, corresponding to cortisol levels lower than 51 nmol/L, equal to 51–138 nmol/L, and higher than 138 nmol/L, respectively.^2^ Indeed, we found that the morning baseline plasma cortisol levels decreased to a greater degree after the 2d‐DST than after the 1 mg‐DST. In the investigation of the cortisol response subgroups, we demonstrated a significantly higher proportion of doubtful or abnormal results after the 1 mg‐DST (63%) than after the 2d‐DST (27%). In other words, the 2d‐DST allowed for the recovery of normal response in more than half of the patients in whom an FP result was obtained following the 1 mg‐DST. To the best of our knowledge, the superiority of the 2d‐DST over the 1 mg‐DST in a healthy population has not been demonstrated till date. However, in our population of healthy women using oral contraceptives, the specificity of the 1 mg‐DST and 2d‐DST is lower (37.0% and 73.1%, respectively) than the reported accuracy of the different tests for the diagnosis of Cushing syndrome showing very high sensitivity and high specificity: for DST [Se 98.6% (96.9%–99.4%) – Spe 90.6% (86.4%–93.6%)], for 2d‐DST [Se 95.3% (91.3%–97.5%) – Spe 92.8% (85.7%–96.5%)] and for LSaC [Se 95.8% (93%–97.2%) – Spe 93.4% (90.7%–95.4%)].^10^ If a DST is considered necessary, our results allow for the use of the 2d‐DST as an alternative to COC discontinuation in women using oral contraceptives.

Another manner in which the problem of high 1 mg‐DST FP rates can be overcome is the accurate evaluation of free cortisol production through the measurement of the levels of salivary cortisol or UFC.

In our study, which enrolled healthy volunteers, we did not observe any abnormal LSaC values when the participants were taking oral contraceptives. We did not observe either any correlation (*r*_s_ = −0.03, *p* = 0.88) between CBG and basal morning salivary cortisol. These results are consistent with those of previous studies, which showed that the level of salivary cortisol does not appear impaired as a result of oral contraception use.^21^ Owing to the extremely low LSaC levels (<2.8 nmol/L), the assay technique we employed was unable to highlight differences in the morning salivary cortisol levels after the 1 mg‐DST and 2d‐DST (<1.9 nmol/L).^22^

To increase the performance of screening tests, it has also been proposed to combine different tests, for example an overnight dexamethasone suppression salivary cortisol test.^23^ Castro et al. have previously reported that the ability to differentiate between obese and CS subjects was improved (with a sensitivity of 100%) by the combination of the late‐night and overnight 1‐mg DST salivary cortisol measurements. More recently, Backlund et al. also showed very high sensitivity and specificity to detect CS with salivary cortisol and cortisone in late‐night samples and after 1 mg‐DST.^24^ These results can only be obtained with techniques allowing the measurement of low concentrations such as mass‐spectrometry assay. Backlund et al. used liquid chromatography‐tandem mass spectrometry to reach, after DST, cortisol and cortisone upper reference limits as low as 0.79 nmol/L and 3.5 nmol/L, respectively. In adrenal incidentaloma, Vieira‐Correa et al. also compare late‐night and 1 mg‐DST saliva with serum cortisol to identify subclinical hypercortisolism.^25^ They conclude that saliva cortisol cannot replace serum cortisol after DST to identify subclinical hypercortisolism in patients with adrenal incidentaloma.

These results confirm the utility of the development of other tools, as the LSaC assay is also far from being easily available. However, a study that used the ERCUSYN database showed that habits have changed in the last 5 years, with testing increasingly being performed on late‐night salivary samples obtained at home.^20^

Currently, UFC measurement is more routinely used than salivary free cortisol measurement. However, the level of UFC may be in the normal range in people with hypercortisolism, justifying the 24‐h collection repetition in those with a strong clinical suspicion of Cushing's syndrome.^8^ Indeed, an intra‐patient variability of 50% has been demonstrated for UFC.^26^ In addition, the level of UFC may be altered by the use of oral contraception or the presence of renal failure.^8^ Additionally, the performance of 24‐h urine collection can prove uncomfortable for participants and the test may be incomplete; therefore, the corresponding results may be non‐interpretative. As a consequence, the level of interest in the DSTs remains high.

In our particular population of women who were taking COCs, the multivariable analysis revealed that two factors were associated with the risk of obtaining FP results: basal plasma cortisol level and COC type. Finally, to specify the population of women in whom the 2d‐DST may be a better option than oral contraceptive use cessation, we expressed the FP percentage for each test in relation to the baseline cortisol value. In our sample, when the basal cortisol level was <700 nmol/L, no FP results were observed after the 2d‐DST; in comparison, after the 1 mg‐DST, 33.3% of the values were <700 nmol/L. When the baseline cortisol level was <900 nmol/L, the efficacy of the 2d‐DST remained clearly stronger, with an FP rate of 5.9%, compared to that of the 1 mg‐DST (50.0%).

## 5 CONCLUSION

Our results confirm that in women taking oral contraceptives, 1 mg‐DST is not accurate as first‐line screening test given its very low specificity. 2d‐DST had higher specificity but remains less accurate than late‐night salivary cortisol. Our results suggest that, when late‐night salivary cortisol is not available, the 2d‐DST could be a better option than the 1 mg‐DST in women using oral contraceptive agents, and the cessation of oral contraception may not always be mandatory for the performance of this test, particularly if the initial cortisol concentration is lower than 900 nmol/L. These conclusions require confirmation in populations with a suspicion of hypercortisolism.

## CONFLICT OF INTEREST

We declare that there is no conflict of interest that could be perceived as prejudicing the impartiality of the research reported.

## Data Availability

In confirm that our article ‘Two‐day low‐dose dexamethasone suppression test more accurate than overnight 1‐mg in women taking oral contraceptives’ with the https://doi.org/10.1002/edm2.255 is a “’contributor‐owned work’ and "all the generated and analysed data presented in this study will be made available. The correspondence should be addressed to N Driessens; Email: natacha.driessens@erasme.ulb.ac.be

## References

[1] van Hulsteijn LT , Pasquali R , Casanueva F , et al. Prevalence of endocrine disorders in obese patients: systematic review and meta‐analysis. Eur J Endocrinol. 2020;182:11‐21. 31652416 10.1530/EJE-19-0666

[2] Fassnacht M , Arlt W , Bancos I , et al. Management of adrenal incidentalomas: European Society of Endocrinology Clinical Practice Guidelines in collaboration with the European Network for the Study of Adrenal Tumors. Eur J Endocrinol. 2016;175:1‐34. 27390021 10.1530/EJE-16-0467

[3] Chiodini I . Diagnosis and treatment of subclinical hypercortisolism. J Clin Endocrinol Metab. 2011;96:1223‐1236. 21367932 10.1210/jc.2010-2722

[4] Reincke M . Subclinical Cushing’s syndrome. Endocrinol Metab Clin North Am. 2000;29:43‐56. 10732263 10.1016/s0889-8529(05)70115-8

[5] Tabarin A . Do the diagnostic criteria for subclinical hypercortisolism exist? Annales d’Endocrinologie. 2018;79:146‐148. 10.1016/j.ando.2018.03.013 29661471

[6] Di Dalmazi G , Vicennati V , Garelli S , et al. Cardiovascular events and mortality in patients with adrenal incidentalomas that are either non‐secreting or associated with intermediate phenotype or subclinical Cushing's syndrome: a 15‐year retrospective study. The Lancet. 2014;2:396‐405. 24795253 10.1016/S2213-8587(13)70211-0

[7] Debono M , Bradburn M , Bull M , Harrison B , Ross RJ , Newell‐Price J . Cortisol as a marker for increased mortality in patients with incidental adrenocortical adenomas. J Clin Endocrinol Metab. 2014;99:4462‐4470. 25238207 10.1210/jc.2014-3007 PMC4255126

[8] Nieman LK , Biller BMK , Findling JW , et al. The diagnosis of Cushing’s syndrome: an Endocrine Society Clinical Practice Guideline. J Clin Endocrinol Metab. 2008;93:1526‐1540. 18334580 10.1210/jc.2008-0125 PMC2386281

[9] Elamin MB , Murad MH , Mullan R , et al. Accuracy of diagnostic tests for Cushing’s syndrome: a systematic review and meta‐analyses. J Clin Endocrinol Metab. 2008;93:1533‐1562. 10.1210/jc.2008-0139 18334594

[10] Galm BP , Qiao N , Klibanski A , Biller BMK , Tritos NA . Accuracy of laboratory tests for the diagnosis of Cushing’s syndrome. J Clin Endocrinol Metab. 2020;105:2081‐2094. 10.1210/clinem/dgaa105 32133504

[11] Meyer EJ , Nenke MA , Rankin W , Lewis JG , Torpy DJ . Corticosteroid‐binding globulin: a review of basic and clinical advances. Horm Metab Res. 2016;48:359‐371. 27214312 10.1055/s-0042-108071

[12] Jung C , Ho JT , Torpy DJ , et al. A longitudinal study of plasma and urinary cortisol in pregnancy and postpartum. J Clin Endocrinol Metab. 2011;96:1533‐1540. 21367926 10.1210/jc.2010-2395

[13] Qureshi AC , Bahri A , Breen LA , et al. The influence of the route of oestrogen administration on serum levels of cortisol‐binding globulin and total cortisol. Clin Endocrinol. 2007;66:632‐635. 10.1111/j.1365-2265.2007.02784.x 17492949

[14] Ruokonen A , Käär K . Effects of desogestrel, levonorgestrel and lynestrenol on serum sex hormone binding globulin, cortisol binding globulin, ceruloplasmin and HDL‐cholesterol. Eur J Obstet Gynecol Reprod Biol. 1985;20:13‐18. 3161762 10.1016/0028-2243(85)90078-4

[15] Paschali M , Willenberg HS , Fritzen R , Schott M , Scherbaum WA , Schinner S . False positives on both dexamethasone testing and urinary free cortisol in women on oral contraception: dose‐response effects. Clin Endocrinol. 2013;79:443‐444. 10.1111/cen.12098 23140463

[16] Ansseau M , Leboulle D , Sulon J , von Frenckell R , Legros JJ . Oral contraceptives and the dexamethasone suppression test. Psychoneuroendocrinology. 1993;18:37‐43. 8475223 10.1016/0306-4530(93)90053-n

[17] Nickelsen T , Lissner W , Schöffling K . The dexamethasone suppression test and long‐term contraceptive treatment: measurement of ACTH or salivary cortisol does not improve the reliability of the test. Exp Clin Endocrinol Diabetes. 1989;94:275‐280. 10.1055/s-0029-1210910 2560985

[18] Vastbinder M , Kuindersma M , Mulder AH , Schuijt MP , Mudde AH . The influence of oral contraceptives on overnight 1 mg dexamethasone suppression test. Netherlands J Med. 2016;74:158‐161. 27185774

[19] Christin‐Maitre S . History of oral contraceptive drugs and their use worldwide. Best Pract Res Clin Endocrinol Metab. 2013;27:3‐12. 23384741 10.1016/j.beem.2012.11.004

[20] Valassi E , Franz H , Brue T , et al. Diagnostic tests for Cushing’s syndrome differ from published guidelines: data from ERCUSYN. Eur J Endocrinol. 2017;176:613‐624. 28377460 10.1530/EJE-16-0967

[21] Manetti L , Rossi G , Grasso L , et al. Usefulness of salivary cortisol in the diagnosis of hypercortisolism: comparison with serum and urinary cortisol. Eur J Endocrinol. 2013;168:315‐321. 23211575 10.1530/EJE-12-0685

[22] Sturmer LR , Dodd D , Chao CS , Shi RZ . Clinical utility of an ultrasensitive late night salivary cortisol assay by tandem mass spectrometry. Steroids. 2018;129:35‐40. 29197558 10.1016/j.steroids.2017.11.014

[23] Castro M , Elias P , Quidute A , Halah F , Moreira A . Out‐patient screening for Cushing’s syndrome: the sensitivity of the combination of circadian rhythm and overnight dexamethasone suppression salivary cortisol tests. J Clin Endocrinol Metab. 1999;84:878‐882. 10084565 10.1210/jcem.84.3.5521

[24] Bäcklund N , Brattsand G , Israelsson M , et al. Reference intervals of salivary cortisol and cortisone and their diagnostic accuracy in Cushing’s syndrome. Eur J Endocrinol. 2020;182:569‐582. 32213657 10.1530/EJE-19-0872

[25] Vieira‐Correa M , Giorgi RB , Oliveira KC , Hayashi LF , Costa‐Barbosa FA , Kater CE . Saliva versus serum cortisol to identify subclinical hypercortisolism in adrenal incidentalomas: simplicity versus accuracy. J Endocrinol Invest. 2019;42:1435‐4442. 31456173 10.1007/s40618-019-01104-8

[26] Petersenn S , Newell‐Price J , Findling JW , et al. High variability in baseline urinary free cortisol values in patients with Cushing’s disease. Clin Endocrinol. 2014;80:261‐269. 10.1111/cen.12259 PMC4231220 23746264

